# A Bayesian approach to detect QTL affecting a simulated binary and quantitative trait

**DOI:** 10.1186/1753-6561-5-S3-S4

**Published:** 2011-05-27

**Authors:** Aniek C Bouwman, Luc LG Janss, Henri CM Heuven

**Affiliations:** 1 Animal Breeding and Genomics Centre, Wageningen University, P.O. Box 338, 6700AH Wageningen, The Netherlands; 2 Aarhus University, DJF Department of Genetics and Biotechnology, P.O. Box 50, 8830 Tjele, Denmark; 3 Clinical Sciences of Companion Animals, Faculty of Veterinary Medicine, Utrecht University, P.O. box 80163, 3508 TD Utrecht, The Netherlands

## Abstract

**Background:**

We analyzed simulated data from the 14^th^ QTL-MAS workshop using a Bayesian approach implemented in the program iBay. The data contained individuals genotypes for 10,031 SNPs and phenotyped for a quantitative and a binary trait.

**Results:**

For the quantitative trait we mapped 8 out of 30 additive QTL, 1 out of 3 imprinted QTL and both epistatic pairs of QTL successfully. For the binary trait we mapped 11 out of 22 additive QTL successfully. Four out of 22 pleiotropic QTL were detected as such.

**Conclusions:**

The Bayesian variable selection method showed to be a successful method for genome-wide association. This method was reasonably fast using dense marker maps.

## Background

Discovering the genetic architecture of traits is not a trivial task, but it is important for our understanding of complex phenotypes. Dense marker maps make it possible to perform genome-wide association (GWA) studies to detect QTL. Bayesian variable selection methods [1] are powerful in association studies, because they can simultaneous take polygenic and all SNP effects into account. This is implemented in packages such as ‘Genomic Selection’ [2] and ‘iBay’ [3]. Meuwissen and Goddard [4] describe how this method could be extended to multi-trait models.

In this paper we analyzed simulated data using a Bayesian approach implemented in the program iBay. The QTL-MAS workshop gives the opportunity to test this method on data with a QTL structure that is unknown beforehand. Although it is hypothesized that the quantitative and binary trait in the dataset are to some degree affected by the same QTL we used an univariate approach because the multivariate version of iBay is still in progress.

## Methods

### Data

The pedigree contained 5 generations, all generations were genotyped but only the first 4 generations (2,326 individuals) were phenotyped for a quantitative and a binary trait. The genome consisted of 5 chromosomes and was genotyped for 10,031 SNPs. A full description of the dataset can be found at the 14^th^ QTL-MAS workshop website [5].

### ASReml analysis

First both traits were analyzed in ASReml [6]. An animal model was applied to estimate the heritability of both traits. A bivariate animal model was applied to estimate the genetic correlation between both traits. In this bivariate analysis the binary trait was analyzed in a linear model. Univariate analysis of the binary trait showed that a linear model gives similar estimates as a threshold model (results not shown).

### QTL analysis

A GWA study was performed on the 2,326 individuals with phenotypes. The data was analyzed with a Bayesian variable selection method [1], implemented in iBay [3]. For QTL detection we used a model that included a polygenic effect as well as all SNPs simultaneously. Variance estimates from ASReml were not used in the model. Sire-dam threshold models are required by iBay to analyze binary traits, therefore the binary trait was analyzed with a sire-dam model, while the quantitative trait was analyzed with an animal model. The following animal model was fitted for the quantitative trait:

and the following threshold sire-dam model was fitted for the binary trait:

where **y** is the quantitative phenotype or the underlying liabilities of the binary phenotype for each individual. Terms  fit marker association effects where  is a vector with allele substitution effects, with *~ N*(0, **I**); **X_k_** is the incidence matrix relating allele substitution effects to observed marker genotypes and  is a scaling factor that shrinks allele effects and models the variance explained by the marker. The scaling factors are conditionally estimated as simple normally distributed regressions, and can be interpreted as a standard deviation. **Z_u_**, **Z_s_** and **Z_d_** are known incidence matrices relating observations to random genetic effects **u**, with , sire **s**, with , and dam **d**, with , respectively. **A** is the numerator relationship matrix, for the sire-dam model the progeny was not included in the relationship matrix. The error vector is , with identity matrix **I**.

In iBay shrinkage of allele effects, through scaling factors *σ_k_*, is done in a dualistic manner by applying a mixture distribution on scaling factors that heavily shrink the effects for most of the markers, effectively removing most of the markers from the model. Only a small part of marker effects are less severely shrunken, identifying markers with important associations. This prior mixture distribution is a mixture of a normal and a truncated normal distribution:

where the first distribution is referred to as the ‘null’ distribution that models the majority of markers with no effect using π_0_ = 0.95 and setting  to a small value. Here  was set to 0.015 for the quantitative trait and to 0.005 for the binary trait (‘null’ markers explain ~2% of phenotypic variation, . The second distribution models markers with important effects. For this second distribution a truncated normal is used so that the signs of estimated allele effects will be identifiable, and the parameter  is estimated from the data, using a flat prior. In this case π_0_/π_1_ was set at 0.95/0.05.

For the mixture prior, the model estimates a ‘mixture indicator’ which indicates for each marker whether it was estimated to belong to the first distribution or the second distribution. The first distribution is indicated by 0 and the second one with 1, so that, after averaging in the MCMC, a value ranging from 0 to 1 which is a posterior probability for each marker to have a large effect (i.e. the probability to belong to the second distribution) and can be used for model selection [1].

### Applied MCMC techniques

All samplers were single site Gibbs samplers. The particular parameterization with scaling factors was chosen so that scaling factors *σ_k_* can be sampled as ‘regressions’ from normal distributions (N(0,1)) and with normal prior distributions.

Multiple MCMC chains of 50,000 cycles with a burn-in period of 1,000 cycles were run until the estimated effective number of samples was >100 for all parameters. The estimated effective number of samples was used as convergence diagnostic based on comparison of within and between chain variances.

### Identification of associated markers

As indicated above, the posterior probability for a marker to come from the second mixture distribution can be used for model selection. We used two approaches to determine a cut-off on these posterior probabilities for the selection of significant associations, denoting the estimated posterior probability by and the prior probabilities used in the model by *π_0_* and *π_1_*.

Analogous to the computation and use of the Bayes Factor between two models we used a ‘parameter-wise Bayes Factor’ (pwBF) as the odds ratio between posterior and prior probabilities for an individual marker:

Using guidelines by Kass and Raftery [7] to judge Bayes Factors, a value above 3.2 is ‘substantial’, a value above 10 is ‘strong’, and a value above 100 is ‘decisive’.

### Post-marker analysis

Using a simultaneous fit of all markers as in the Bayesian variable selection method can cause the signal of a QTL to be spread over multiple markers. In that case individual marker have a moderate posterior probability, but the group of markers has a high joint posterior probability. The primary joint Gibbs samples for the mixture indicators were used, which take account of the switches for adjacent markers being on or off, to derive the joint probability for having a signal in a window. Different grouping-windows with size of 1 up to and including 11 SNP in a window were tried on the output. First, a probability for the presence of a QTL at all is given. Secondly, if there is a QTL present in the window, the probability of multiple QTL in the window is given. If the mixture indicators show that more than one SNP within a window has a high probability of being in the model, this is counted to determine the probability of multiple QTL.

## Results

### ASReml

Table 1 shows phenotypic variance and genetic parameters for both traits. The bivariate analysis of the traits showed a positive genetic correlation of 0.66 between the traits.

**Table 1:** Phenotypic and genetic parameters for the quantitative (Q) and binary (B) trait.

Trait	Phenotypic variance	Genetic parameters^a^	
Q	104.35	0.54	
B	0.21	0.66	0.23

^a^ Heritabilities are on the diagonal and genetic correlation below the diagonal.

### iBay

**Figure 1:**
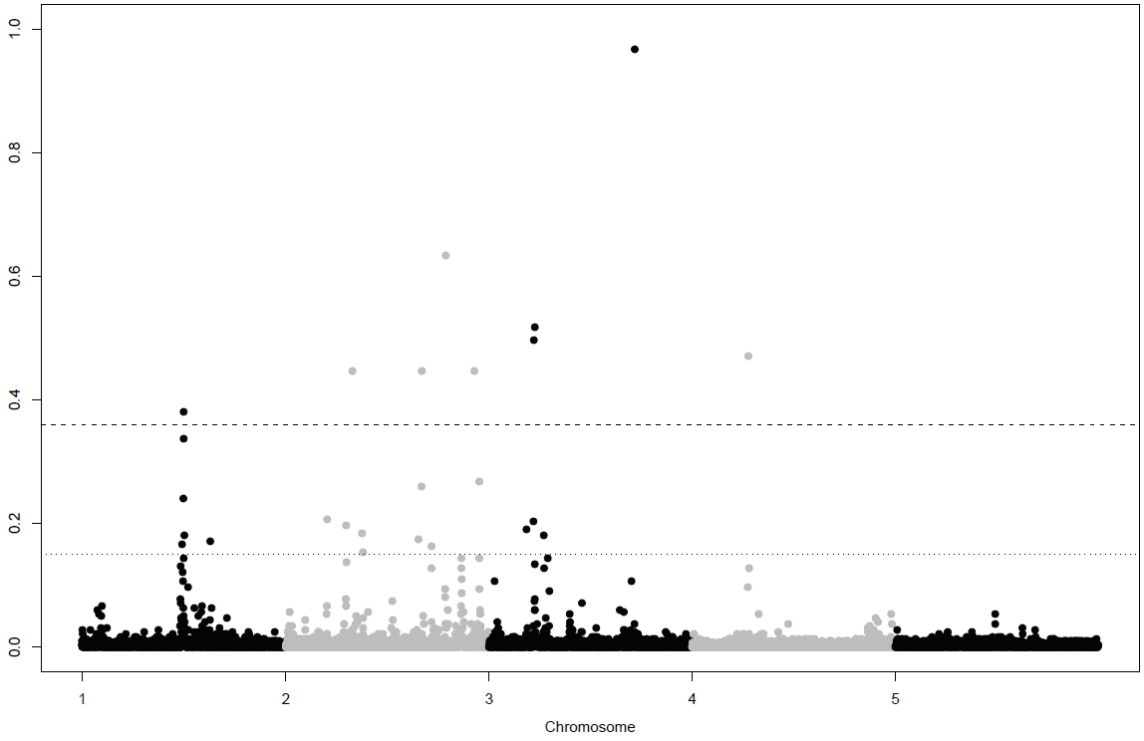
**Manhattanplot of posterior probabilities for the quantitative trait.** Dashed and dotted lines are thresholds for significant and putative levels at parameter-wise Bayes Factor of 10 and 3.2 respectively.

**Figure 2:**
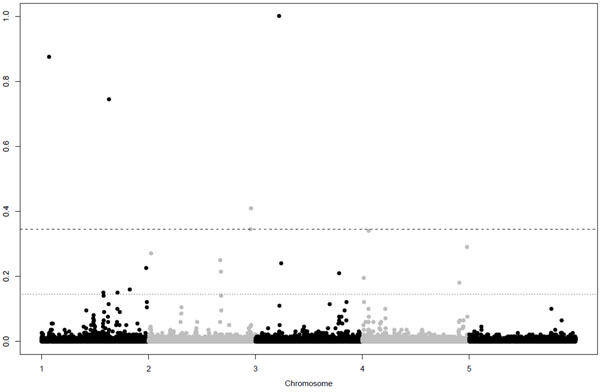
**Manhattanplot of posterior probabilities for the binary trait.** Dashed and dotted lines are thresholds for significant and putative levels at Bayes Factor of 10 and 3.2 respectively.

GWA for the quantitative trait resulted in 9 significant and 16 putative SNPs, the GWA for the binary trait resulted in 5 significant and 13 putative SNPs (Table 2). Figure 1 and 2 show Manhattan plots for the quantitative and binary trait respectively. For both traits QTL were detected on all chromosomes, except chromosome 5, were none were simulated. Successfully mapped QTL are given in Table 3, next to the simulated details of these QTL. Mainly QTL with large effects were detected. Among the significant SNPs there was only one false positive, indicating that our threshold was conservative, but could make a good distinction between significant and putative QTL.

**Table 2:** Loci associated with the quantitative (Q) and binary (B) trait, their parameter-wise Bayes Factor (pwBF) and posterior probability (Prob(2ndMix))

Trait	Locus	Chr	Position	pwBF	Prob(2ndMix)
Q	5488	3	71,610,807	551.0	0.97
	3623	2	78,604,040	32.8	0.63
	4485	3	22,443,619	20.3	0.52
	4480	3	22,030,629	18.7	0.50
	6703	4	27,663,560	16.8	0.47
	2719	2	32,741,451	15.3	0.45
	3405	2	66,759,090	15.3	0.45
	3905	2	92,573,498	15.3	0.45
	954	1	50,009,335	11.6	0.38
	952	1	49,965,266	9.6	0.34
	3948	2	94,982,901	6.9	0.27
	3402	2	66,632,577	6.7	0.26
	947	1	49,825,082	6.0	0.24
	2465	2	20,369,230	4.9	0.21
	4477	3	21,919,975	4.8	0.20
	2658	2	29,667,353	4.7	0.20
	4411	3	18,509,382	4.5	0.19
	2810	2	37,448,320	4.3	0.18
	4559	3	26,890,769	4.2	0.18
	959	1	50,316,379	4.2	0.18
	3381	2	65,270,284	4.0	0.17
	1215	1	63,017,238	3.9	0.17
	939	1	49,185,089	3.8	0.17
	3498	2	71,583,451	3.7	0.16
	2827	2	37,933,865	3.4	0.15
B	4480	3	22,030,629	1881.0	1.00
	145	1	7,149,725	133.0	0.88
	1215	1	63,017,238	55.5	0.75
	3961	2	95,493,425	13.2	0.41
	3948	2	94,982,901	10.0	0.34
	6217	4	5,977,635	9.8	0.34
	8030	4	97,774,814	7.8	0.29
	2033	2	2,213,453	7.0	0.27
	3405	2	66,759,090	6.3	0.25
	4511	3	23,981,734	6.0	0.24
	1913	1	97,688,161	5.5	0.23
	3421	2	67,468,328	5.2	0.22
	5616	3	78,155,543	5.1	0.21
	6127	4	1,456,752	4.6	0.19
	7887	4	90,517,506	4.2	0.18
	1631	1	82,409,839	3.6	0.16
	1102	1	57,850,647	3.4	0.15
	1383	1	70,982,584	3.4	0.15

**Table 3:** Comparison simulated and detected QTL for the quantitative (Q) and binary (B) trait.

Simulated						Detected^c^			
Q	B				var	Q		B	
						
QTL	QTL	Chr	Position	SNP^b^	QTL	SNP	Position	SNP	Position

1	*	1	7,536,081	R152	1.84			145	7,149,725
2	*	1	50,389,545	R960	1.13	959	50,316,379		
3	*	1	58,038,782	R1106	1.09			1102	57,850,647
4	*	1	63,386,317	L1226	1.19	1215	63,017,238	1215	63,017,238

5	*	2	2,289,495	R2036	0.97			2033	2,213,453
6	*	2	30,511,220	L2675	0.48	2658	29,667,353		
8	*	2	67,248,417	L3414	0.87	3405	66,759,090	3421	67,468,328
11	*	2	94,680,408	L3946	0.40	3948	94,982,901	3948	94,982,901
12	*	2	95,449,160	R3959	1.13			3961	95,493,425

14	*	3	22,415,527	L4483	4.50	4485	22,443,619	4480	22,030,629
17^a^		3	71,610,807	5488	4.49	5488	71,610,807		
18	*	3	78,153,081	R5616	0.29			5616	78,155,543

22	*	4	6,296,223	R6224	0.57			6217	5,977,635
24		4	26,749,857	R6684	0.14	6703	27,663,560		
30	*	4	97,651,414	R8024	0.72			8030	97,774,814

31^a^	Epi	1	49,185,089	939	7.01	939	79,185,089		
32^a^	Epi	1	50,316,379	959		959	50,316,379		

33^a^	Epi	2	32,617,381	2715	4.18	2719	32,741,451		
34^a^	Epi	2	33,139,075	2727		2719	32,741,451		

36^a^	Imp	2	78,604,040	3623	2.20	3623	78,604,040		

* QTL had pleiotropic effect (affected also binary trait)

Epi: epistatic QTL pair 1 (31-32) and 2 (33-34), only affecting the quantitative trait

Imp: paternally imprinted QTL, only affecting the quantitative trait

^a^ QTL was on chip

^b^ Closest SNP to the right (R) or left (L) of the QTL

^c^ QTL were considered detected if the position was within 1Mbp from the simulated QTL.

Table 4 shows post-marker analysis results for both traits. Post-marker analysis showed that some regions had a probability of more than one QTL in the region.

**Table 4:** Post-marker analysis of the quantitative (Q) and binary (B) trait

Trait	Region Size ^a^	Pr(≥1) ^b^	Pr(>1)^c^	Marker start	Marker end
Q	5	1.00	0.25	946	950
	1	1.00	0.00	5488	5488
	6	0.96	0.11	4479	4484
	10	0.78	0.22	951	960
	10	0.78	0.15	3901	3910
	5	0.76	0.25	4485	4489
	9	0.60	0.00	6696	6704
B	1	1.00	0.00	4480	4480
	3	1.00	0.19	4482	4484
	9	0.88	0.05	137	145
	10	0.86	0.08	1207	1216

^a^ different grouping-windows with size of 1 up to and including 11 SNP were analyzed, region size is the number of SNPs in the window

^b^ probability of presence of a QTL in the region

^c^ probability of more than one QTL in case there was a QTL present in the region

### Pleiotropy

Four QTL were segregating in both traits (Table 5). Pleiotropic effects of these QTL explained only 10% of the genetic correlation between the traits by including the SNPs as fixed effects in the bivariate animal model in ASReml (results not shown).

**Table 5:** Pleiotropic SNPs and their parameter-wise Bayes Factors (pwBF) for the quantitative (Q) and binary (B) trait

SNP	Chr	Position	Q pwBF	B pwBF
4480	3	22,030,629	18.7	1881.0
3405	2	66,759,090	15.3	6.3
3948	2	94,982,901	6.9	10.0
1215	1	63,017,238	3.9	55.5

## Discussion

The technique used by iBay are a Bayesian hierarchical regression model similar to Bayesian Lasso, by introduction of a variance parameter per marker, and a model using a mixture model following the version of the Bayesian variable selection method by George and McCullogh [1]. The SNP variance originates from a mixture of two distributions, one for the SNP with an effect on the phenotype and the other for SNPs without an effect on the phenotype. The method is similar to BayesB [8]. However, BayesB uses an informative prior which is estimated from the data, in contrast iBay uses a fixed prior.

For the quantitative trait we ran 6 MCMC chains of 50,000 cycles with a burn-in period of 1,000 cycles. One chain took approximately 2.5 hour on a dual core Intel 2.33 GHz processor, so in total it took 15 hours. For the binary trait only 4 MCMC chains were needed, which took 10 hours.

A univariate QTL analysis was performed on the simulated data. However, a multivariate QTL analysis would increase the power and the precision of the pleiotropic QTL position [9,10]. Multivariate analysis is especially beneficial when one of the traits has a low heritability [10]. The simulated data contained two traits with relatively high heritabilities, therefore, the univariate analysis was able to detect the main QTL for either trait. A multivariate analysis might be able to detect the pleiotropic QTL with small effects as well.

## Conclusions

The Bayesian variable selection method showed to be a successful method for GWA. This method was reasonably fast using dense marker maps. The univariate Bayesian analysis was able to detect the main QTL, however, a multivariate approach might be able to detect more pleiotropic QTL and to a more precise position.

## Competing interests

The authors declare that they have no competing interests.

## Authors' contributions

ACB analyzed data and wrote manuscript. LLGJ developed software (iBay), participated in project design and coordination. HCMH conceived project, participated in project design, coordination and revising the manuscript.
